# Readiness and Implementation of Evidence-Based Practice Among Physiotherapists: A Cross-Sectional Study and Evidence-Based Practice Questionnaire Validation

**DOI:** 10.3390/jcm15051716

**Published:** 2026-02-24

**Authors:** Christi Ojaste, Jarek Mäestu, Kadri Medijainen

**Affiliations:** 1 Institute of Sport Sciences and Physiotherapy, Faculty of Medicine, University of Tartu, Estonia, Ujula 4, 51008 Tartu, Estonia; jarek.maestu@ut.ee (J.M.); kadri.medijainen@ut.ee (K.M.); 2 Department of Rehabilitation, Rakvere Hospital, Estonia, Lõuna põik 1, 44316 Rakvere, Estonia

**Keywords:** evidence-based physiotherapy, physical therapists, EBPQ, implementation, competence, psychometrics

## Abstract

**Background/Objectives**: Evidence-based practice (EBP) is a core competence in physiotherapy, yet its implementation in routine clinical practice remains inconsistent. This study aimed to describe self-reported EBP competencies among physiotherapists and to examine factors associated with daily EBP and readiness to implement EBP. **Methods**: A cross-sectional analytic survey was conducted among 337 practicing Estonian physiotherapists (75% female) between 2022 and 2024. EBP competencies were assessed using the Estonian version of the Evidence-Based Practice Questionnaire (EBPQ-E). Two multiple linear regression models examined associations of demographic and professional characteristics and EBP competencies with (1) daily EBP and (2) readiness to implement EBP. **Results**: The mean total EBPQ-E score was 4.72 (SD = 0.89), with the highest scores in *Attitude*, followed by *Knowledge/Skills*, *Practice*, and *Sharing*. While physiotherapists strongly endorsed the value of EBP, critical appraisal and knowledge sharing were less frequent. Readiness to implement EBP was associated with supervisory experience, dual employment, and working with colleagues (*p* < 0.05), explaining 7.5% of the variance. Daily EBP was primarily explained by EBP competencies (40.8% variance), whereas 5–10 years of qualification showed a negative association. **Conclusions**: While professional and contextual factors support readiness for EBP, sustained implementation relies on continuous competency development and career-long support for practicing physiotherapists, shifting focus away from static background characteristics, workload, and time constraints.

## 1. Introduction

Evidence-based practice (EBP) integrates clinical expertise with the best available research evidence through systematic processes—ask, acquire, appraise, apply, assess—applicable across all healthcare fields [1]. Mastery of EBP requires lifelong, self-directed learning encompassing up to 68 competencies [2] and is essential for safe, high-quality care by reducing misuse, overuse, and underuse of services [3], including in physiotherapy [4].

Despite its recognized value, studies report a persistent gap between research and practice among physiotherapists, with EBP only modestly integrated into routine care [5,6,7,8]. There is still no unified approach to improving the implementation of EBP [9]. The literature indicates that the main barriers relate to professionals’ insufficient research-related knowledge and skills, as well as organizational characteristics of the work environment, with lack of time being the most frequently cited constraint [7,8,9,10,11]. Heterogeneous assessment tools and analyses hinder reliable comparisons [11,12], and no universal gold standard exists [13]. The Evidence-Based Practice Questionnaire (EBPQ) [14] is the most widely used standardized measure, initially designed for nurses but validated across professions [15]. To date, the EBPQ has not been applied to physiotherapists as a sufficiently large independent sample; in a study using a mixed healthcare professional sample, physiotherapists constituted only a small proportion of participants (12%) [16]. Furthermore, a validated Estonian-language version of the EBPQ is currently not available.

Professional competence in EBP involves both valuing its importance and possessing the skills for its effective implementation [17]. Yet, evidence on strategies to integrate EBP into physiotherapists’ daily practice remains limited. The aims of the study included (1) evaluating the internal reliability and construct validity of the Estonian EBPQ; (2) describing physiotherapists’ self-assessed EBP competencies; and (3) examining how demographic and professional characteristics, and EBP competencies relate to daily EBP and general readiness for EBP implementation.

## 2. Materials and Methods

### 2.1. Study Design

This observational quantitative online survey followed a repeated cross-sectional design and adhered to the STROBE (Strengthening the Reporting of Observational Studies in Epidemiology) guidelines [18].

### 2.2. Setting, Participants, and Sample Size

The study was conducted within Estonia’s publicly funded, solidarity-based healthcare system. As of 2024, an estimated 850 physiotherapists (82% female) were practicing in the country [19], while roughly 1350 individuals held a physiotherapy qualification, indicating that not all qualified professionals were engaged in clinical practice at the time of the study. Physiotherapists work in both public and private sectors, as employees or employers, and generally operate a high degree of professional autonomy in clinical decision-making, irrespective of practice setting. Although a physiotherapy qualification permits lifelong practice without re-certification, the study population was restricted to physiotherapists engaged in daily clinical practice to avoid excessive heterogeneity and ensure the validity of the questionnaire. Proficiency in Estonian, the official language of education and healthcare, was required for eligibility. The study sample exhibited characteristics comparable to those reported in previous research [5,7,20].

Inclusion criteria were: (1) at least entry-level higher education in physiotherapy or an equivalent qualification, (2) active employment as a physiotherapist, and (3) proficiency in Estonian to complete the questionnaire. Sample size estimation using G*Power 3.1.9.7 (Heinrich-Heine-Universität Düsseldorf, Düsseldorf, Germany) [21] indicated a minimum of 252 participants to achieve 95% power (α = 0.05) for detecting medium effect sizes in the planned analyses.

### 2.3. Instrument

The Evidence-Based Practice Questionnaire (EBPQ) [14] assesses self-reported daily EBP, attitudes toward EBP, and related knowledge/skills, providing an overall indication of an individual’s readiness to implement EBP. It consists of 24 items across three subscales: *Practice* (6 items), *Attitude* (4 pairs of contrasting items), and *Knowledge/Skills* (14 items). Items are rated on a 7-point Likert scale (1 = never/do not agree/poor; 7 = frequently/strongly agree/really good), with higher scores indicating a greater engagement in EBP.

In this paper, the 24-item EBPQ was translated into Estonian using independent forward and backward translation, ensuring content validity. Face validity was confirmed in a pilot study. Minor adjustments were made to clarify terminology and enhance construct validity. Exploratory factor analysis yielded factor loadings ranging from 0.28 to 0.83. One item with a loading below 0.30 was removed in accordance with established criteria and considering the sample size [22], resulting in a revised 23-item, four-factor questionnaire. All subsequent analyses were conducted using this version, which possessed optimal psychometric properties, including internal consistency (Table 1).

### 2.4. Data Collection

There is no official registry of practicing physiotherapists in Estonia, and membership of a professional association is not mandatory. Therefore, a convenience sampling strategy was employed, recruiting participants via multiple channels, including publicly available contacts and organizational networks, to maximize reach among qualified physiotherapists, following Dillman’s method [23]. The survey was conducted across three distinct periods using the LimeSurvey platform, version en-1.0.0 (free software under the GNU General Public License v2 or later), with reminder invitations sent three weeks after the initial invitation.

### 2.5. Statistical Analysis

**Figure 1:**
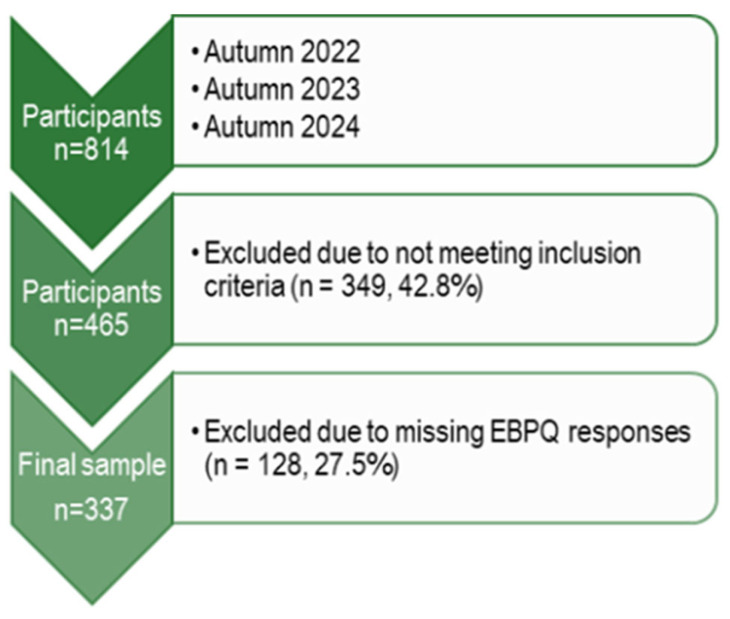
Flow diagram of participant recruitment, exclusions at each stage, and final study sample.

Data exported from LimeSurvey to Microsoft Excel 365 (Microsoft Corporation, Redmond, WA, USA; version 2312), where responses were assessed for eligibility based on the predefined inclusion criteria (Figure 1). Statistical analyses were performed in R (R Core Team, Vienna, Austria; version 2024.09.1). Quantitative variables were summarized using means and standard deviations, and categorical variables were summarized by frequency. Normality was assessed using Shapiro–Wilk tests and histograms, which guided the choice of parametric (*t*-test, Analysis of Variance (ANOVA)) or non-parametric (Mann–Whitney U, Kruskal–Wallis) tests. For comparisons involving three groups, if a statistically significant difference was observed in ANOVA or Kruskal–Wallis tests, Bonferroni-corrected post hoc analyses were conducted, and the post hoc results are presented in Table 2 as *p*-values (*p* < 0.05). Multiple linear regression was used to examine the influence of background variables and self-assessed competencies on daily EBP, as well as the effect of background variables on overall readiness to implement EBP. The assumptions of linearity, independence, homoscedasticity, and normality of residuals were verified before conducting the regression analyses. A linear relationship was identified between age group and professional qualification; consequently, professional qualification was selected for inclusion in the model.

## 3. Results

### 3.1. Sample Characteristics

Altogether, this study analysed the responses of 337 respondents, representing 38% of physiotherapists working in Estonia. A chi-square test comparing the sample’s gender distribution (75% women) with the national population (82% women) indicated minor deviations (χ^2^ = 11.53, *p* < 0.001), suggesting the sample is broadly representative. Respondents were predominantly women with entry-level education and considered themselves specialized in a specific field of physiotherapy. Part-time and full-time (or more) workloads were equally represented, with a daily average of 5–10 patients treated (Table 2).

**Table 2:** Background characteristics of respondents and statistically significant between-group differences in EBPQ-E total and subscale mean scores.

Characteristic	Division	N (%)	N/A (%)	Practice	Attitude	Knowledge/Skills	Sharing	Total
Gender	male female	82 (24) 252 (75)	3 (1)	-	-	**	-	*
Age ^1^	20–29	150 (45)	1 (0)	-	*	-	-	-
	30–39	132 (39)						
	≥40	54 (16)						
Highest professional education	entry level Master’s degree	206 (61) 131 (39)	0 (0)	-	-	-	*	-
Professional qualification ^2^ (years)	<5	148 (44)	1 (0)	-	-	-	**	-
	5–10	84 (25)						
	>10	104 (31)						
Specialization	yes no	221 (66) 108 (32)	8 (2)	-	-	*	**	*
Employment ^3^	salaried employee	229 (68)	4 (1)	*	-	**	**	**
	self-employed	33 (10)						
	dual employment	71 (21)						
Workload	part-time full-time or more	157 (47) 180 (53)	0 (0)	-	-	-	-	-
Patient load per day	<5	43 (13)	1 (0)	-	-	-	-	-
	5–10	234 (69)						
	>10	59 (18)						
Supervising experience	yes no	214 (63) 122 (37)	1 (0)	-	**	*	***	**

Fellowphysiotherapist	yes no	286 (85) 47 (14)	4 (1)	*	-	*	*	*


N/A—not answered; * *p* < 0.05; ** *p* < 0.01; *** *p* < 0.001, - = not significant; ^1^ post hoc test indicates 20–29 > 30–39; ^2^ post hoc test indicates >10 > <5; ^3^ post hoc test indicates dual employment > salaried employee.

### 3.2. Group Differences in Self-Assessed Readiness to Implement EBP by Background Variables

The mean total EBPQ-E score exceeded the scale midpoint (Likert 4) in all comparison groups (detailed results presented in Supplementary Table S1). Significant differences emerged for professional characteristics—specialization, supervisory experience, employment status, and the presence of physiotherapist colleagues—as well as for the demographic characteristics of gender in total scores and in several competence subscales. Physiotherapists with dual employment demonstrated higher EBPQ-E scores compared with those employed solely in employed roles, as did those with supervisory experience, male physiotherapists, individuals with a specialization, and those working alongside fellow physiotherapists. Beyond the previously examined comparison groups, additional subscale-specific differences emerged: on the *Attitude* subscale, physiotherapists aged 20–29 scored significantly higher than those aged 30–39, and on the *Sharing* subscale, respondents with a Master’s degree scored higher than entry-level physiotherapists, as did those with more than ten years of qualification compared with those with less than five years. In contrast, no statistically significant differences were observed in any subscale or total EBPQ-E scores across levels of patient load or workload.

### 3.3. Physiotherapists’ Self-Assessed Competencies

Overall, physiotherapists reported stronger attitudes and greater knowledge and skills related to EBP than they actually applied in their daily clinical work (Table 3). The only item with a mean score below the scale midpoint (Likert 4) related to the critical appraisal of literature, corresponding to the third step of the EBP process. Items on informing colleagues and spreading new ideas, as well as assessing validity and evaluating evidence critically, received scores near the midpoint, reflecting lower confidence in sharing competencies and knowledge and skills. The greatest variability (SD ≥ 1.5) was observed in items from the *Sharing* and *Practice* subscales, particularly in those items with the lowest mean scores. Statements on attitudes toward EBP received the highest ratings, exceeding a score of 5, and research skills also surpassed this threshold. Research skills, along with identifying practice gaps and reflecting on practice, were rated highly, reflecting greater confidence in these EBP-related knowledge and skills.

### 3.4. Multiple Linear Regression Analyses for Daily Practice and Overall Readiness to Implement EBP

The EBPQ-E model explained 7.5% of the variance in readiness to implement EBP (Table 4). Supervisory experience, dual employment, and the confirmed existence of a fellow physiotherapist were positively associated with higher readiness. The Practice model explained 40.8% of the variance in daily EBP use, with higher scores on *Knowledge/Skills*, *Attitude*, and *Sharing* positively associated with higher *Practice* subscale scores. Negatively associated with 5 to 10 years of qualification as a physiotherapist.

## 4. Discussion

This study represents the first comprehensive evaluation of physiotherapists’ self-assessed EBP competencies using the EBPQ, analyzed item by item, offering a novel perspective on their readiness to implement EBP and providing new insights that enable the identification of specific steps in the five-step EBP process and facilitate targeted improvement strategies. Examining specific skills and knowledge offers more informative insights than assessing overall competency, thereby guiding practical strategies in clinical settings. The sample closely reflected the demographic and professional characteristics of Estonian physiotherapists [19], supporting national representativeness. Predominantly female, mainly entry-level educated, and treating 5–10 patients daily, the sample is comparable to physiotherapist populations in countries with well-established professional and research infrastructures (Sweden, Austria, Canada, Italy, Australia, UAE, Jordan, Kuwait) [5,7,17,20,24,25,26], whereas studies from countries with less developed infrastructures (Cameroon, Sri Lanka, Kenya, Ecuador, Viet Nam, Colombia) [8,27,28,29,30] report higher proportions of male physiotherapists and higher patient loads, highlighting demographic and organizational differences. This comparability supports the study’s external validity, indicating that the findings can be generalized while accounting for demographic, professional, and contextual characteristics.

In relation to the first study objective, the Estonian version of the questionnaire demonstrated acceptable psychometric properties after modifications to ensure construct validity, including the removal of one *Attitude* item and restructuring into a four-factor model, in line with earlier findings [16,31,32,33,34]. Overall, the adapted questionnaire, including the *Sharing* subscale, provides a more comprehensive assessment of EBP by capturing the essential role of professional interaction [7,29] and peer influence among physiotherapists [6,9,29,35]. Updating and standardizing the instrument could enable cross-disciplinary use and provide a reliable tool for monitoring and enhancing competencies, ultimately supporting improved patient care and outcomes.

In accordance with the study’s second aim, physiotherapists generally reported a positive attitude toward EBP and confidence in searching for scientific sources, while expressing considerable uncertainty in critically appraising evidence, consistent with previous studies among physiotherapists [8,17,20,24,25,27,28] and among nurses [36]. However, substantial variability was observed in the *Practice* and *Sharing* subscales. Item-level analysis indicated that the greatest variability was related to critical appraisal skills, which also emerged as the most variable step in the EBP process within the *Practice* subscale. The high variability in *Practice* scores suggests inconsistent implementation of EBP in daily clinical practice, whereas variability in the *Sharing* subscale is likely influenced by the availability of colleagues and opportunities for professional interaction. Within the Theory of Planned Behavior [37], attitude appears consistently positive among physiotherapists [10,17,20,26,38] and can therefore be considered a fulfilled prerequisite for intention formation. Subjective norm is closely linked to colleagues’ influence, an aspect that has yielded mixed findings in previous research [6,9,29,35], while at a broader level, there is a clear societal expectation that healthcare services be evidence-based [3]. In contrast, perceived behavioral control shows notable variability. Over time, recurring personal skill gaps and organizational constraints have been observed [10,11]. Overall, the discrepancy between positive attitudes and practice indicates an implementation challenge in which intentions are constrained by social and organizational contexts, explaining the limited or inconsistent application of EBP in physiotherapy practice. To address this implementation gap, critical appraisal should be treated as a shared interprofessional competence, with system-level support and structured interprofessional interaction embedded in the clinical environment to enable collective skill development across allied health professions.

Addressing the third aim, our findings indicate that while specific background characteristics—such as specialization, male gender, supervisory experience, peer support, and dual employment—are associated with higher self-assessed EBP-related competencies, consistent with previous studies [5,7,26,30], their relevance for day-to-day EBP appears to be limited. Similar to the results reported by Alqahtani et al. [39] among nurses, background characteristics explained only a small proportion of the variance in readiness to implement EBP. In contrast, competency-related factors accounted for a substantially larger share of daily EBP implementation. Apart from a negative association observed among physiotherapists with 5–10 years of experience, who reported less frequent EBP implementation compared with both less and more experienced colleagues, demographic and work-related factors showed little explanatory value. This finding echoes the inconsistent and sometimes contradictory evidence reported in earlier research [5,20,40]. Career-stage factors, including work–life balance and family responsibilities, may influence engagement and the adoption of evidence-based practice; however, a negative correlation between career plateau and work engagement has been reported among nurses [41], yet remains largely unexplored among physiotherapists. Overall, these findings suggest that individual competencies, rather than demographic or professional characteristics, are the primary drivers of EBP engagement in everyday clinical practice. It underscores the need for future research to focus on underexplored influences, including personality and motivation, as well as gender differences, and to examine whether clinical challenges stimulate EBP engagement or merely reflect the characteristics of more proactive individuals.

Although previous research suggests that EBP engagement varies by educational level [5,7,20,28]. Instead, they indicate that neither advanced education nor workload meaningfully differentiates self-assessed EBP competencies in daily clinical practice. This finding should also be interpreted in the light of the fact that the vast majority of practicing physiotherapists, both in prior research [5,8,27,42] and in the present study, hold entry-level qualifications. Within a solidarity-based healthcare system, where physiotherapists with European Qualifications Framework levels 6 and 7 [43] when performing comparable roles and carrying similar responsibilities, this distribution may further attenuate observable differences related to degree level. Notably, despite the continued flexibility and variation in entry-level education in Europe (3–5 years) [44], our results revealed no significant differences in EBP readiness or implementation between shorter and longer educational pathways. This may partly reflect the ongoing evolution of entry-level curricula, which increasingly emphasize EBP compared with earlier cohorts. Consistent with previous reports that identify high workload and limited time as barriers to EBP implementation [6,25,26,29], our results suggest that physiotherapists maintain comparable EBP competence despite working under demanding conditions. Overall, these findings shift the focus from structural factors, such as degree level or workload, to how professional development and workplace support mechanisms sustain EBP standards in clinical settings. This perspective highlights the potential importance of professional development structures, such as voluntary membership in professional associations.

Several limitations should be considered when interpreting the findings of this study. First, questionnaire responses may be influenced by social desirability bias, whereby participants select responses they perceive as more socially acceptable. This is a common and inherent limitation of self-reported data. Second, using a web-based survey introduces the potential for self-selection bias, as individuals who are more digitally proficient or more confident in their evidence-based practice competencies may have been more likely to participate. Additionally, the cross-sectional design limits the ability to infer causality and captures competencies simultaneously, rather than allowing for longitudinal assessment. Despite these limitations, a key strength of the study lies in the sample’s representativeness in terms of size and demographic diversity, as well as the high statistical power of the analyses, which together enhance the generalizability and robustness of the findings to the broader physiotherapy population.

## 5. Conclusions

The Estonian EBPQ demonstrated acceptable psychometric properties after modifications, providing a comprehensive assessment of EBP, including professional interaction and peer influence. Physiotherapists reported positive attitudes and confidence in searching for evidence, but showed considerable variability in their critical appraisal skills, consistent with the process of daily practice implementation and with appraising the literature. Demographic and professional characteristics had limited influence, whereas individual competencies were the primary drivers of EBP implementation. Differences in years of clinical experience highlight the need to harmonize EBP competencies across the career span, ensuring that practicing physiotherapists receive support and guidance to enhance their daily EBP implementation. Overall, these findings highlight the need for systematic, multi-level strategies to support consistent EBP implementation in clinical practice and underscore the importance of exploring additional influences that may more strongly affect EBP in clinical practice.

## Figures and Tables

**Table 1:** Validity and reliability indices for the 3- and 4-factor questionnaire models.

Model Fit Indices ^1^	3-Factor, 24 Items	4-Factor, 23 Items
χ2/df	4.81	4.16
CFI	0.79	0.84
TLI	0.77	0.82
RMSEA	0.106	0.097
[95% CI]	[0.100–0.112]	[0.090–0.103]
SRMR	0.08	0.07
**Reliability ^2^**		
Practice	α = 0.85 (IT = 0.51–0.79)	α = 0.85 (IT = 0.60–0.78)
Attitude	α = 0.69 (IT = 0.32–0.51)	α = 0.68 (IT = 0.42–0.57)
Knowledge/Skills	α = 0.92 (IT = 0.40–0.78)	α = 0.91 (IT = 0.42–0.79)
Sharing	-	α = 0.82 (IT = 0.66–0.74)
Total	α = 0.93	α = 0.93

^1^ χ^2^/df = chi-square test, CFI = comparative fit index, TLI = Tucker–Lewis index, RMSEA = root mean square error of approximation, 95% CI = 95% confidence interval, SRMR = standardized root mean square residual; ^2^ α = Cronbach’ s α, IT = item-total correlation.

**Table 3:** Scores of each EBPQ-E item, its subscales, and the entire questionnaire (N = 337).

Subscale	Item	Mean (SD)	Rank Order
*Practice*	Formulating clear questions	4.50 (1.63)	17
4.52 (1.31)	Finding relevant evidence	4.70 (1.60)	15
	Critically appraising literature	3.79 (1.75)	23
	Integrating evidence with expertise	4.83 (1.50)	7–8
	Assessing outcomes	4.79 (1.65)	11–13
*Attitude*	Encouraging feedback	5.50 (1.37)	2
5.70 (0.95)	Evidence-based practice is essential	6.20 (1.00)	1
	Adapting practice based on evidence	5.39 (1.27)	3
*Knowledge* */Skills*	Research skills Information Technology (IT) skills Auditing practice Formulating research questions Knowing key info sources Spotting practice gaps Retrieving evidence Evaluating evidence critically Assessing validity Assessing clinical relevance Personalizing information Reflecting on practice	5.08 (1.20) 4.79 (1.36) 4.80 (1.14) 4.30 (1.29) 4.64 (1.24) 4.88 (1.18) 4.83 (1.27) 4.14 (1.46) 4.22 (1.44) 4.75 (1.32) 4.80 (1.27) 4.84 (1.28)	4 11–13 9–10 18 16 5 7–8 20 19 14 9–10 6
4.67 (0.94)			









*Sharing*	Informing colleagues	4.01 (1.92)	22
4.28 (1.51)	Exchanging ideas	4.79 (1.61)	11–13
	Spreading new ideas	4.04 (1.68)	21
**Total**			
4.72 (0.89)			

SD—standard deviation.

**Table 4:** Multiple linear regression analysis results for two models.

Independent Variable		B-Coefficients	Standardised β	*p*	Model Statistics
EBPQ-E (N = 321)					
Gender	male	0.22	0.11	0.056	R^2^ = 0.075 F (12, 304) = 3.13 *p* < 0.001
Education	Master’s	0.14	0.08	0.214	
Specialized	yes	0.15	0.08	0.156	
Workload	full or more	0.01	0.01	0.908	
Supervising	yes	0.31	0.17	**0.007**	
Patient load	5–10	0.02	0.01	0.913	
	>10	0.02	0.01	0.924	
Qualification	5–10 >10	−0.19	−0.09	0.170	
		−0.23	−0.12	0.111	
Employment	dual self-employed	0.43	0.19	**0.001**	
		0.12	0.04	0.498	
Fellow physiotherapist	yes	0.33	0.13	**0.025**	
Practice (N = 321)					
Gender	male	−0.04	−0.01	0.757	R^2^ = 0.408 F (15, 301) = 15.49 *p* < 0.001
Education	Master’s	−0.14	−0.05	0.311	
Specialized	yes	−0.01	0.00	0.926	
Workload	full or more	0.18	0.07	0.156	
Supervising	yes	0.07	0.03	0.586	
Patient load	5–10 >10	0.21	0.08	0.249	
		0.20	0.06	0.397	
Qualification	5–10 >10	−0.32	−0.11	**0.043**	
		−0.04	−0.01	0.836	
Employment	dual self-employed	0.16	0.05	0.320	
		0.01	0.00	0.980	
Fellow physiotherapist	yes	0.10	0.03	0.549	
*Knowledge/Skills*		0.58	0.42	**<0.001**	
*Attitude*		0.34	0.24	**<0.001**	
*Sharing*		0.10	0.12	**0.028**	

Reference categories: female, entry level, not specialized, part-time workload, no supervising experience, < 5 patients per day, < 5 years qualification, salaried employee, the presence of a fellow physiotherapist was not confirmed; N = number of respondents in each model; cases with missing predictor data were excluded, R^2^ = adjusted coefficient of determination, F = statistic for ANOVA; in bold *p*-values < 0.05.

## Data Availability

Due to restrictions imposed by the ethics committee, the data underlying this study cannot be made publicly available, but may be obtained from the corresponding author upon reasonable request.

## Supplementary Materials

The following supporting information can be downloaded at: https://www.mdpi.com/article/10.3390/jcm15051716/s1, Table S1: Mean subscale and total EBPQ-E scores by background and professional characteristics.

## Abbreviations

The following abbreviations are used in this manuscript:

STROBE	Strengthening the Reporting of Observational Studies in Epidemiology
EBP	Evidence-Based Practice
EBPQ	Evidence-Based Practice Questionnaire
EBPQ-E	The Estonian version of the Evidence-Based Practice Questionnaire
