# Supplementary material for: Readiness and Implementation of Evidence-Based Practice Among Physiotherapists: A Cross-Sectional Study and Evidence-Based Practice Questionnaire Validation
Source: J Clin Med. 2026 Feb 24;15(5):1716. doi: 10.3390/jcm15051716 (PMC12985529; doi:10.3390/jcm15051716)
Supplement: Supplementary file 1 [file jcm-15-01716-s001.zip › jcm-4115390-supplementary.pdf]

Supplemental Table S1. Mean subscale and total EBPQ-E scores by background and professional characteristics.

**Characteristic**	**Mean score** (SD)				
	Practice	Attitude	Knowledge/Skills	Sharing	Total
**Gender** (N=334)					
male (n=82)	4.71 (1.23)	5.72 (1.03)	4.91 (0.97)	4.51 (1.49)	4.94 (0.90)
female (n=252)	4.44 (1.32)	67 (0.92)	4.59 (0.91)	4.19 (1.50)	4.66 (0.88)
	p = 0.084†	p = 0.695†	**p = 0.009†**	p = 0.093†	**p = 0.014†**
**Age group** (N=336)					
20-29 (n=150)	4.55 (1.16)	5.63 (0.78)	4.62 (0.85)	4.08 (1.41)	4.70 (0.80)
30-39 (n=132)	4.50 (1.43)	5.85 (0.92)	4.74 (1.02)	4.49 (1.52)	4.80 (0.97)
≥ 40 (n=54)	4.43 (1.41)	5.45 (1.31)	4.62 (0.97)	4.25 (1.68)	4.66 (0.96)
	p = 0.988‡	**p = 0.024‡**	p = 0.517†	p = 0.061‡	p = 0.518†
**Qualification** (N=336)					
< 5 (n=148)	4.60 (1.18)	5.67 (0.85)	4.65 (0.84)	3.99 (1.44)	4.73 (0.79)
5-10 (n=84)	4.31 (1.39)	5.70 (0.84)	4.69 (1.00)	4.33 (1.49)	4.69 (0.95)
>10 (n=104)	4.58 (1.41)	5.71 (1.15)	4.69 (1.04)	4.61 (1.55)	4.79 (0.99)
	p = 0.300‡	p = 0.459‡	p = 0.912†	**p = 0.003‡**	p = 0.742†
**Education** (N=337)					
entry level (n=206)	4.53 (1.30)	5.65 (0.88)	4.61 (0.88)	4.11 (1.48)	4.70 (0.85)
Master's degree (n=131)	4.50 (1.31)	5.76 (1.05)	4.76 (1.02)	4.53 (1.51)	4.80 (0.96)
	p = 0.888‡	p = 0.088‡	p = 0.177†	**p = 0.014‡**	p = 0.320†
**Specialization** (N=329)					
yes (n=221)	4.60 (1.29)	5.76 (0.95)	4.75 (0.94)	4.45 (1.47)	4.82 (0.90)
no (n=108)	4.37 (1.33)	5.56 (0.91)	4.53 (0.92)	3.90 (1.51)	4.59 (0.84)
	p = 0.190‡	p = 0.052‡	**p = 0.037‡**	**p = 0.002‡**	**p = 0.020†**
**Employment** (N=333)					
employed (n=229)	4.47 (1.25)	5.68 (0.86)	4.58 (0.92)	4.08 (1.50)	4.66 (0.87)
self-employed (n=33)	4.26 (1.37)	5.42 (1.33)	4.67 (0.93)	4.52 (1.61)	4.64 (0.88)
dual (n=71)	4.83 (1.40)	5.87 (0.98)	4.99 (0.97)	4.77 (1.39)	5.05 (0.94)
	**p = 0.031‡**	p = 0.115‡	**p = 0.006†**	**p = 0.002‡**	**p = 0.005†**
**Workload** (N=337)					
part-time (n=157)	4.39 (1.30)	5.70 (0.93)	4.68 (0.88)	4.27 (1.51)	4.70 (0.84)
full-time or more (n=180)	4.63 (1.31)	5.69 (0.97)	4.67 (0.99)	4.27 (1.50)	4.77 (0.94)
	p = 0.113‡	p = 0.908‡	p = 0.988†	p = 0.978‡	p = 0.479†
**Patient load** (N=336)					
< 5 (n=43)	4.28 (1.26)	5.57 (1.22)	4.78 (0.85)	4.47 (1.49)	4.71 (0.81)
5-10 (n=234)	4.53 (1.32)	5.71 (0.90)	4.64 (0.94)	4.21 (1.54)	4.73 (0.91)
>10 (n=59)	4.64 (1.29)	5.70 (0.90)	4.72 (1.00)	4.36 (1.41)	4.81 (0.92)
	p = 0.384‡	p = 0.927‡	p = 0.860†	p = 0.565‡	p = 0.884‡
**Supervising** (N=336)					
yes (n=214)	4.62 (1.27)	5.78 (1.00)	4.75 (0.95)	4.49 (1.47)	4.83 (0.88)
no (n=122)	4.32 (1.35)	5.53 (0.81)	4.52 (0.89)	3.88 (1.48)	4.56 (0.87)
	p = 0.076‡	**p = 0.001‡**	**p = 0.025†**	**p < 0.001‡**	**p = 0.006†**
**Fellow physiotherapist** (N=333)					

yes (n=286)	4.61 (1.27)	5.72 (0.97)	4.72 (0.94)	4.36 (1.51)	4.80 (0.89)
no (n=47)	4.12 (1.36)	5.60 (0.74)	4.41 (0.88)	3.79 (1.45)	4.45 (0.84)
	**p = 0.019**‡	p = 0.182‡	**p = 0.030**†	**p = 0.026**‡	**p = 0.011**†

SD = standard deviation, in bold, p values < 0.05.

†t-test for two-group comparisons; ANOVA for three-group comparisons.

‡Mann–Whitney U test for two-group comparisons; Kruskal–Wallis test for three-group comparisons.
